# Anticancer traits of chimeric antigen receptors (CARs)-Natural Killer (NK) cells as novel approaches for melanoma treatment

**DOI:** 10.1186/s12885-022-10320-0

**Published:** 2022-11-25

**Authors:** Maryam Bahmanyar, Mohammad Kazem Vakil, Ghaidaa Raheem Lateef Al-Awsi, Seyed Amin Kouhpayeh, Yaser Mansoori, Behnam Mansoori, Ali Moravej, Abdulbaset Mazarzaei, Abdolmajid Ghasemian

**Affiliations:** 1 Noncommunicable Diseases Research Center, Fasa University of Medical Sciences, Fasa, Iran; 2 Department of Radiological Techniques, Al-Mustaqbal University College, Babylon, Iraq; 3 Department of Pharmacology, Faculty of Medicine, Fasa University of Medical Sciences, Fasa, Iran; 4 Department of Immunology, Iranshahr University of Medical Sciences, Iranshahr, Iran

**Keywords:** Chimeric antigen receptors, Natural killer cells, CAR NK cells, Melanoma, Combination therapies

## Abstract

Owing to non-responsiveness of a high number of patients to the common melanoma therapies, seeking novel approaches seem as an unmet requirement. Chimeric antigen receptor (CAR) T cells were initially employed against recurrent or refractory B cell malignancies. However, advanced stages or pretreated patients have insufficient T cells (lymphopenia) amount for collection and clinical application. Additionally, this process is time-consuming and logistically cumbersome. Another limitation of this approach is toxicity and cytokine release syndrome (CRS) progress and neurotoxicity syndrome (NS). Natural killer (NK) cells are a versatile component of the innate immunity and have several advantages over T cells in the application for therapies such as availability, unique biological features, safety profile, cost effectiveness and higher tissue residence. Additionally, CAR NK cells do not develop Graft-versus-host disease (GvHD) and are independent of host HLA genotype. Notably, the NK cells number and activity is affected in the tumor microenvironment (TME), paving the way for developing novel approaches by enhancing their maturation and functionality. The CAR NK cells short lifespan is a double edge sword declining toxicity and reducing their persistence. Bispecific and Trispecific Killer Cell Engagers (BiKE and Trike, respectively) are emerging and promising immunotherapies for efficient antibody dependent cell cytotoxicity (ADCC). CAR NK cells have some limitations in terms of expanding and transducing NK cells from donors to achieve clinical response. Clinical trials are in scarcity regarding the CAR NK cell-based cancer therapies. The CAR NK cells short life span following irradiation before infusion limits their efficiency inhibiting their in vivo expansion. The CAR NK cells efficacy enhancement in terms of lifespan TME preparation and stability is a goal for melanoma treatment. Combination therapies using CAR NK cells and chemotherapy can also overcome therapy limitations.

## Background

**Fig. 1:**
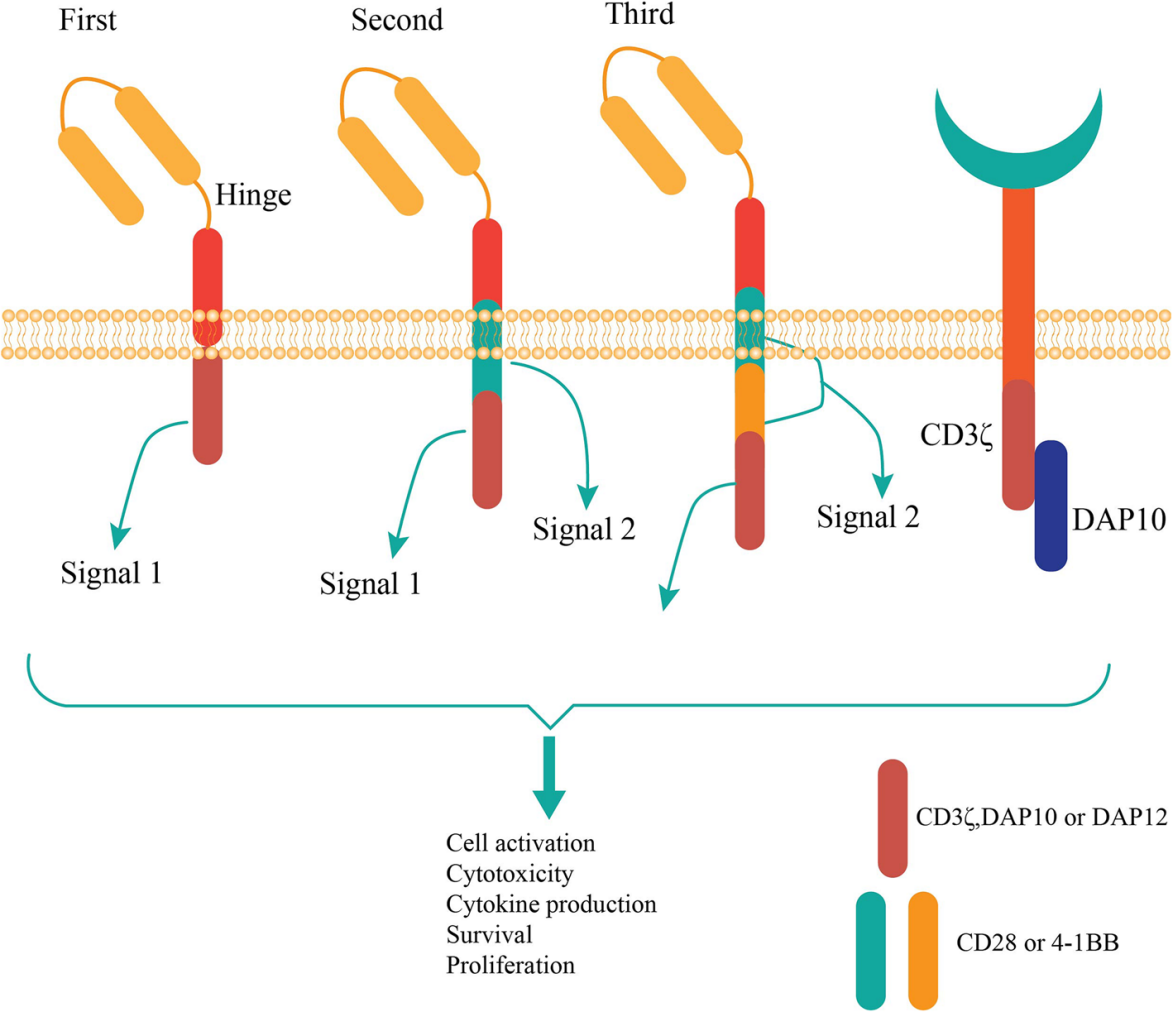
Detailed effects of CAR construct in signaling; the CAR construct includes first, second and third generations with gradual improvement of function. Various signals from CAR molecules lead to the NK cell activation, cytotoxicity, cytokine production, survival and proliferation

Melanoma is among three skin cancers (squamous and basal cell carcinoma) with the highest metastatic potential and mortality rate [1, 2]. Although several drugs such as Ipilimumab (anti-CTLA-4) and Nivolumab (anti-PD1) checkpoint antibodies are applied, a high number of patients still do not response to these agents [3, 4]. Chimeric antigen receptors (CARs) T cells have been applied for relapsed malignancies [5, 6]. Nonetheless, some limitations in autologous and allogeneic settings are remained to be solved [7, 8]. Natural killer (NK) cells have several advantages over T cells in the application for therapies such as availability, unique biological features, safety profile, cost effectiveness and more prevalent existence in tissues [9–12]. In this method, personal cells are taken, engineered to synthesize specific receptors and expanded ex vivo and infused into the same patient as immunotherapy. The extracellular part of CAR includes single chain antigen-specific variable light and heavy chain antibodies and the intracellular domain comprises a signaling molecule from the cell receptor. More advanced CAR molecules also contain co-stimulatory molecules (nanobodies, CD28, designed ankyrin repeat proteins or DARPins, 4-1BB or CD137, CD3ζ or cytokines) in the intracellular domain (Fig. 1). CAR T cells were initially employed against recurrent or refractory B cell malignancies [13, 14]. However, advanced stages or pretreated patients have insufficient T cells (lymphopenia) amount for collection and clinically application.

Additionally, this process is time-consuming and logistically cumbersome. Another limitation of this approach is toxicity and cytokine release syndrome (CRS) progress and neurotoxicity syndrome (NS) [8, 15, 16]. Considering these, circumventing of limitations can be accomplished using alternative CAR NK cells. As CD3-CD56+ innate lymphoid cells, NK cells play a substantial antimicrobial and anticancer role. Inadvertently, NK cells killing effect against transformed cells is independent of antigen priming, major histocompatibility complex molecules and target cells expression [17–19]. After insertion of CAR construct into the T or NK cell genome, it is expressed onto the cell surface. The cells are expanded and injected into the cancer patient. CAR can recognize the tumor cells and destruct them. Despite advantages of alternative CAR NK cells versus CAR T cells, few clinical trials have been performed with this regard. As the innate immune part, NK cells intrinsically recognize absence of human leukocyte antigen (HLA)-proteins which overcome escape mechanisms by cancer cells. Several studies have deciphered that CAR NK cell based anticancer therapies have been highly efficient and rapid with lower costs than those of CAR T cells against melanoma [20–24].

### NK cells and cancer

NK cells are a versatile component of the innate immunity, divided into two cytokine-producing CD56^bright^ and CD56^dim^CD16+ cells [25]. These cells constitute only 5–15% of total NK cells and contain innate ability to recognize transformed cells [26–28]. NK cells have various stimulatory and inhibitory receptors playing a critical role in the elimination of infected, stressed, foreign and cancer cells by activating other immune cells such as dendritic cells, B cells and T cells and production of pro-inflammatory cytokines [26, 29–31]. These cells have various inhibitory and activator receptors. NK cells produce indispensable levels of INFγ and in NK cells deficient conditions, cure from several tumor cell lines has been difficult [32, 33]. Moreover, pre-activated murine NK cells combined with radiotherapy has more efficiently decreased cancer cells [34]. In melanoma patients, NK cells have been existed in peripheral blood as well as in tissues with variable results. However, CD56^bright^ and CD56^dim^ NK cells subsets may remain without alterations in metastatic melanoma patients blood, but their function may be impaired such as in degranulation, and production of IFNγ and NKG2D [35]. The clinical outcome of several tumors has been associated with NK cells primary infiltration and activation. CD57 + KIR+CD56^dim^ NK cells as entirely mature and effector cells play an important role in melanoma cells combating [36, 37]. In lymph nodes, NK cells with high level expression of NKp46, CD16, NKG2D, NKp44, DNAM-1 and NKp30 has been found [38–40]. The efficacy of NK cells based therapies against solid tumors in controversial [41, 42]. One strategy was to electroporation of these cells with two receptor-specific mRNA constructs (CXCR-1 and NKG2D) into the tumor microenvironment (TME) of peritoneal ovarian cancer xenografts in mice [43, 44]. As antibody-dependent cytotoxic cells, NK cells interact with α-CTLA-4 antibody which in turn activate these cells [45, 46]. A recent clinical trial on 29 late stage (III/IV) melanoma patients unraveled that NK cells subsets were similar in rate among healthy and patients and demonstrated low rate of CD56^bright^ NK cell subsets in treated melanoma patients [35]. Low CD56^bright^ NK cell frequency in melanoma patients treated with Ipilimumab was considered as a good result of survival. These cells inhibit the T cell responses via CD38, perforin, CD11a and IFNγ [47, 48]. Importantly, immune cells such as NK cells may deplete in nutrients and exhaust for efficient anticancer cytotoxicity in the TME. Indeed, various melanoma cells lines response diversely to NK cell-mediated killing due to the diverse expression of various proteins [49]. Hence, providing required nutrients is necessary. In addition to the cytotoxicity against melanoma cells, NK cells promote their recruitment via High Mobility Group Box-1 (HMGB1) protein [50]. Melanoma cells mainly express NKG2D and DNAM-1 ligands but not ULBPs or nectin-2, which suggests that NK cells are activated via these receptors against melanoma [51, 52]. Other receptors such as MHC class I chain related-proteins A (MICA) and B (MICB) or MICA/B, NKp30, NKp44, and NKp46 have been also expressed in melanoma cell lines [53, 54]. Lower number of CD56^bright^ NK cells in stage IV of melanoma can be a prognostic factor and a biomarker. Notably, the NK cells number and activity is affected in the TME, paving the way for developing novel approaches by enhancing their maturation and functionality.

### Immune escape mechanisms by melanoma cells

In the primary stage of cancer, various immune cells participate in the elimination of melanoma cells, however, high plasticity of tumor cells leads to immune evasion [55, 56]. A mutation in v-raf murine sarcoma viral oncogene homolog (*BRAF)*-E600 gene occurred in about 40–50% of melanoma patients causes resistance to monotherapy [57, 58]. In spite of expression of several ligands for NK cells by various cancer cells, melanoma cells escape responses via cytokines production, immunosuppressive cells activation, lower MHC expression and creation of hypoxic tumor microenvironment [59–61]. Various inhibitory ligands such as HLA-E, galectin − 9, PD-L1, CD155 and CD 112 are expressed by melanoma cells [62–66]. Furthermore, immunosuppressive cytokines and molecules such as adenosine, indoleamine 2,3-dioxygenase (IDO), matrix metalloproteinases (MMPs), vascular endothelial growth factor (VEGF), arginase-1 (ARG-1), IL-10 and tumor growth factor-β (TGF-β) are employed to inactivate the NK cells [67–69]. The hypoxic conditions created by melanoma cells alter the immune cells activities by expression of HIF-1α. These conditions cause [70, 71] the autophagy induction in the NK cells and mitigates their responses to cytokines such as IL-2, IL-12, IL-15 and IL-21, thereby inhibiting natural cytotoxicity triggering receptors (NCRs) and NKG2D NK cells activating receptors. Hypoxia also alters the expression of cancer cells ligands.

### Sources for NK cells expanding

Seeking and finding those suitable sources for NK cells obtainment and expansion is important. NK cells are derived from peripheral blood mononuclear cells (PBMCs), umbilical cord blood (UCB), bone marrow (BM) (healthy or patient-derived), induced pluripotent stem (iPS) cell and immortalized NK cell lines [72]. NK-92 cell line is a proper source for convenient and sufficient expanding of NK cells [73]. Additionally, high cytotoxicity and safety of NK-92 cells (irradiated before clinical use) has been verified in preclinical and clinical studies. These cells also lack NKp44 and NKp46 NK cells activating receptors [74, 75]. Other NK cell lines such as KHYG-1, NKL, NKG, NK-YS, NK-L, NK 3.3, EP3138905A1 and YT have been applied for expanding [76, 77]. K562 cell line with increased activity has also been developed [78, 79]. A wide anticancer cytotoxicity has been observed using CAR NK cells derived from KHYG-1 cells [80]. Due to the probability of activation and proliferation of regulatory cells, proper procedure for NK cells expanding seems essential. In addition, genetically modified NK cells to improve their function, persistence and capacity include introduction of genes into NK cells (IL-2 and IL-15 coding genes), CARs, NKG2A inhibitory receptor downregulation, viral transduction, transfection and mRNA electroporation [81, 82]. Regarding clinical trials, Anti-CD33 CAR NK cells for acute myeloid leukemia (NCT02944162, PMID: 28054442), Allogeneic anti-CD19 CAR NK cells for CD19 + Leukemia (NCT02892695, PMID: 28054442), ROBO1 CAR NK cells for Solid tumor expressing ROBO1 (NCT03940820) and allogeneic anti-MUC1 CAR pNK cells for MUC1-positive solid tumor (NCT02839954) have been performed.

### Immunotherapies using NK cells

**Fig. 2:**
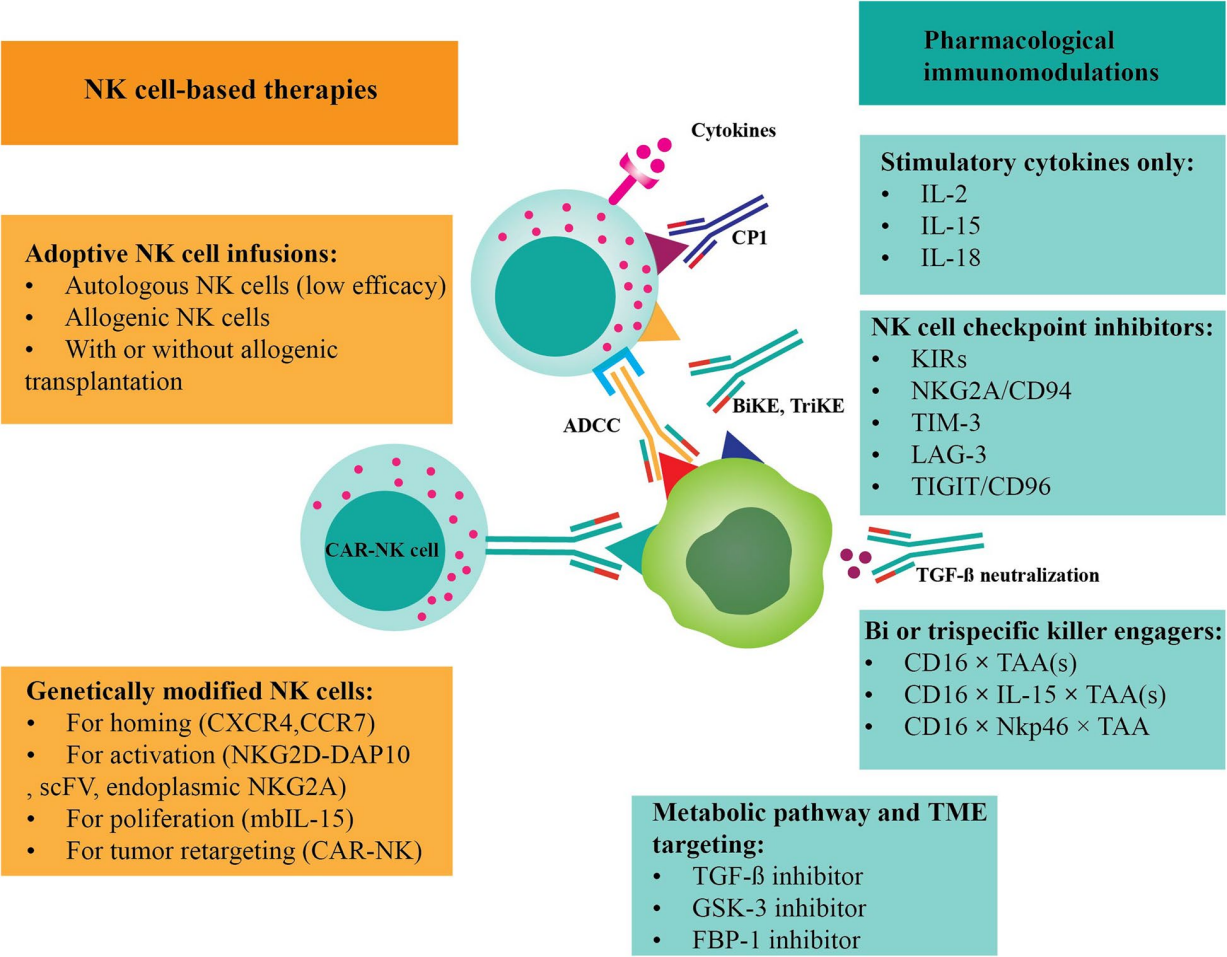
Various therapeutic approaches to enhance the anticancer effects of NK cells; allogenic NK cells are developed for infusions, and genetically modified NK cells which include those for homing (CXCR4, CCR7), for activation (NKG2D-DAP10, scFV and endoplasmic NKG2A), for proliferation (mbIL-15) and for tumor retargeting (CAR-NK cells)

**Fig. 3:**
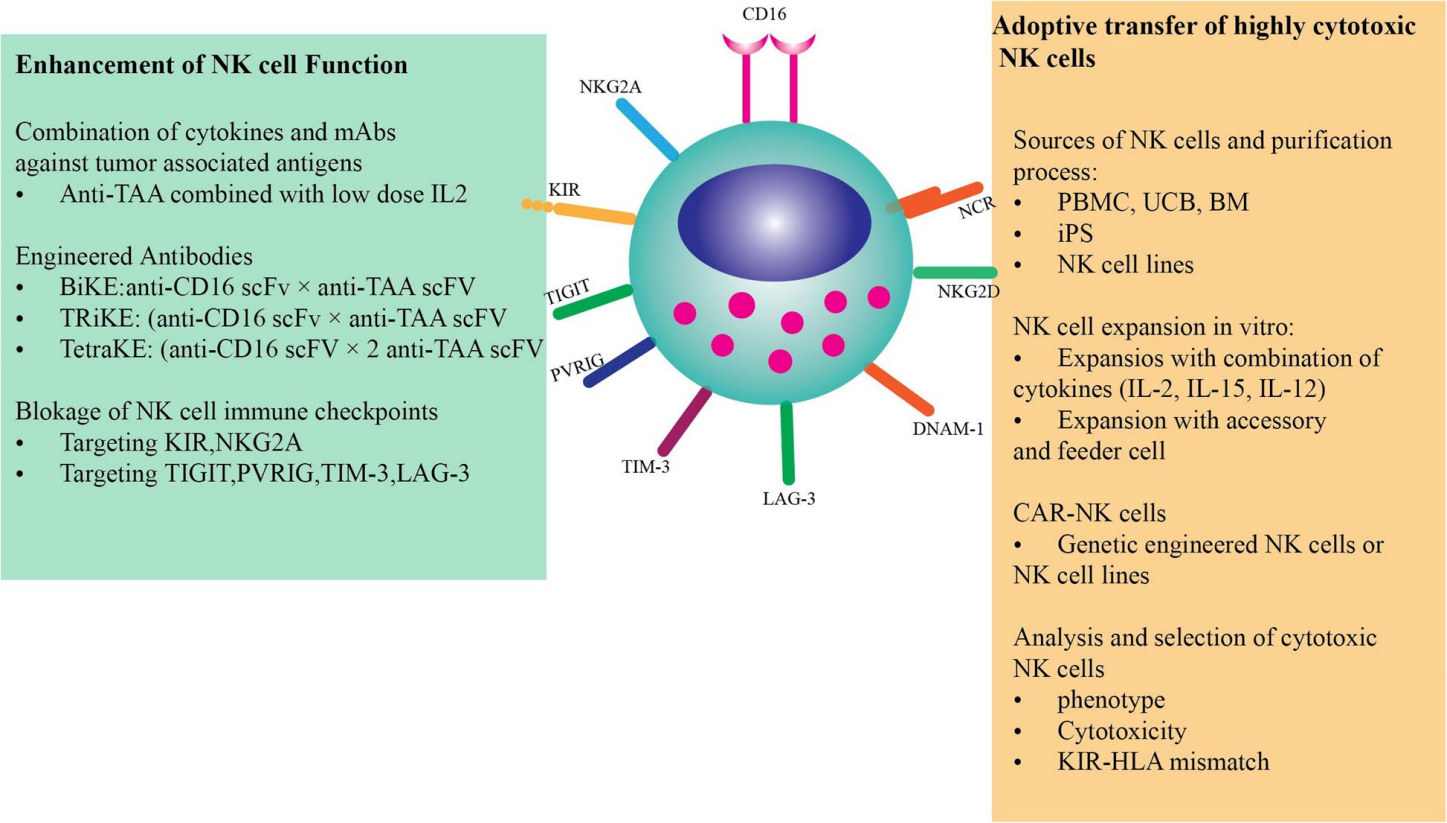
NK cells cancer immunotherapy enhancement via receptors modification; NKG2A: natural killer cell group 2 member A, KIR: Killer cell immunoglobulin-like receptors, TIGIT: T cell immunoreceptor with Ig and ITIM domains, PVRIG: PVR Related Immunoglobulin Domain Containing, TIM-3: T cell immunoglobulin and mucin domain-containing protein 3, LAG-3: Lymphocyte Activating 3, DNAM-1: DNAX Accessory Molecule-1, NCR: Natural Cytotoxicity Triggering Receptor 1

NK cells have infiltrated in various cancers differently. Targeting CTLA-4 and PD-1 checkpoint inhibitors (CPIs) have affected the function of T cells and NK cells in cancer treatment [83–85]. CD56^dim^ NK cells express PD-1 as a differently expressed ligand on the NK cells [86]. Those NK cells expressing PD-1 can improve functionality (cytotoxicity and granzyme + perforin production) following PD-1 blockade [87, 88]. Interestingly, NK and CD8+ T cells cooperate in melanoma and tumors cells. Blockade of other inhibitory receptors such as KIR, NKG2A, T cell immunoglobulin and mucin domain-containing protein 3 (TIM-3) and T cell immunoreceptor with Ig and ITIM domains (TIGIT) have been also demonstrated [89, 90]. Hence, activated NK cells release granzyme B and perforin. Other therapies have included cytokine (IL-2, IL-15) therapy, oncolytic viruses and specific antibodies (Figs. 2 and 3) [91, 92]. CD16 receptor plays a substantial role in NK cells activity. However, variations in allotype of CD16 lead to different affinity of antibodies for appropriate antibody dependent cell cytotoxicity (ADCC) induction of NK cells [93–95]. Bispecific and Trispecific Killer Cell Engagers (BiKE and Trike, respectively) are emerging and promising immunotherapies for efficient ADCC by NK cells [96, 97]. BiKEs include two single chain variable fragments (scFvs) of antibody each specific for CD16 or antigen connected by a flexible linker. TriKEs have an additional antigen specific chain or cytokine (IL-15). IL-15 has higher efficiency and lower toxicity than IL-2 for NK cells activation [98, 99]. Furthermore, TriKEs are more efficient in NK cells activation and effector function (cytotoxicity and interferon gamma/INFγ and tumor necrosis factor alpha/TNFα production) [100]. However, not all cancer cells express antigens and therefore, expression of virus antigens by BiKE and Trike constructs will be promising for melanoma cells targeting. Talimogene laherparepvec (T-VEC) is a herpes simplex virus-1 (HSV-1) applied against melanoma cells [101, 102]. These antigens are not expressed by human cells, hence utilization of BiKE and Trike for their expression is promising as safer and more efficient immunotherapy. CAR NK cells recognize tumor antigens not only by CAR, but also through their own receptors. Indeed, a balance of inhibitory or provoking signals determines the NK cell function. The enhancement of safety and efficacy of CAR NK therapy can be achieved through some strategies such as integration of suicide genes and silencing NK inhibitory receptors (IL-4 and IL-7 receptor) [103, 104]. CAR NK cells have some limitations in terms of expanding and transducing NK cells from donors to achieve clinical response. Obtaining high number of NK cells can be achieved by feeder-free, bovine serum-free protocol [105]. CAR NK cells have shown proper anticancer effect by lower level production of INFγ and TNFα. Nevertheless, clinical efficacy and infusion persistence within TME is still low using CAR NK cells monotherapy against solid tumors despite sufficient safety.

### Combinatorial strategies against melanoma using NK cells

There is a scarcity of data regarding CAR NK cell-based combination therapies for the melanoma. CAR NK cell therapy has unraveled great breakthrough for the treatment of cancers, however, its efficacy is limited for solid tumors. Hence utilizing combination therapies with chemical anticancer drugs is warranted. Regarding melanoma, no previous combination therapies of CAR NK cells and chemical agents has been investigated. It was demonstrated that synergistic use of OV-IL15C plus epidermal growth factor (EGFR)-CAR NK cells was able to suppress glioblastoma (GBM) cells and also enhanced the infiltration of NK and CD8^+^ T cells [106]. Another study exhibited that EpCAM-CAR NK-92 cells and Regorafenib could synergistically prohibit the colorectal cancer cells in vitro and in vivo exhibiting a significantly higher efficacy than monotherapy using CAR NK cells. Additionally, Bortezomib- modified oncolytic viruses (OV) combined with the NK cell infusion therapy exhibited more efficient anticancer effects [107, 108].

### Limitations of NK cells-based immunotherapies

The requirement for cytokine support for NK cells activation is an important limitation for successful clinical therapy despite their benefits for long-term low toxicity [26, 109]. In addition, the use of IL-2 and IL-15 cytokines for the proliferation of NK cells is associated with toxicity and neutropenia. Moreover, IL-2 activates the regulatory T (T_reg_) cells which inhibit the NK cells activation [110]. To overcome this problem, IL-2-diphteria toxin and lymphodepleting agents have been applied prior to the infusion of NK cells [111]. Furthermore, TME leads to inhibition of NK cells by some factors such as T_regs_, MDSCs, tumor growth factor-β (TGF-β), CD20, HLA-E, galactin-9 and CD8 [31, 112–114]. It was exhibited that viral transduction leads to higher NK cells apoptosis compared to the CAR T cells.

### CAR NK cells therapies against melanoma

There is a scarcity of data regarding the applications of CAR NK cells against melanoma (Fig. 3). Given the success of CAR NK cells in hematological cancer, their application in solid tumors is warranted. Solid tumors develop resistance against drugs and escape immune cells. CAR NK cells CD7-CAR NK-92MI and dCD7-CAR NK-92MI cells have outlined efficient anti-cancer effects in vitro and in vivo against T-leukaemia cell lines [115]. Moreover, CAR.CD19-CD28-zeta-2A-iC9-IL-15-transduced HLA-mismatched CB NK cells were applied for patients with CD19+ B-lymphoid malignancies [116]. CAR NK cells have been also employed against myeloma, lymphoma, ovarian cancer, glioblastoma, colorectal cancer, breast cancer, lung cancer, pancreatic cancer, glioma, prostatic and gastric cancer [31, 117–119]. Previous CAR NK cell therapies against melanoma have targeted GPA7 and CD276 (B7-H3) using NK-92 as the NK cell source [24, 28]. Additionally, the vectors included lentivirus and retrovirus. Additionally, the CAR construction has been His-tag and F (ab)2 and HLA-A2TM + CD3ζ. The CAR NK cell therapies are still in primary stages and combination therapies with adoptive cell therapies and immune checkpoint inhibitors (ICKs) will be promising. One limitation of CAR NK cell therapy is their short life span for irradiation before infusion which unable them for multiplication in vivo. Anti-CD19 car NK cells were applied for chronic lymphocytic leukemia and lymphoma in Phase I clinical trial. NK-92 cells targeting Her2 in solid tumors (NCT04050709), Indeed, NK cells need cytokine support for persistence in the body. Additionally, lack expression of natural cytotoxicity receptors (NCRs) and CD16, tumorigenesis of NK-92 cells and stroke risk include concerns about the CAR NK cells application [120]. CAR NK cells low penetration into the TME is also a limitation [121]. NK cells only develop memory cells against viruses not for cancer [9].

### Future prospects

The improvement of CAR NK cells in terms of preparation, stability and lifespan and penetration into the TME is necessary for efficient application for approval of them in cancer therapy. Novel CAR macrophages which abundantly infiltrate into the TME is promising for solid tumor therapy. Combination therapies using CAR NK cells and chemotherapy can also overcome limitations.

## Conclusion

The transforming of NK cells into potent tumor killing cells is crucial in melanoma treatment owing to high immunogenicity of melanoma cells. NK cells are versatile multifunctional immune cells which efficiently participate in anticancer activities in cooperation with CD8+ and CD4+ T cells. The therapeutic failure of CAR T cells in the TME is mainly due to the lack of CAR T cells specific tumor antigens which inhibit these cells. CAR NK cells have advantages over CAR T cells in terms of natural cytotoxic capacity, lack of off-target toxicity, low costs, ready availability and convenient antitumor activation by antigens both dependent and independent of MHC molecules. Short life span of CAR NK cells is advantageous for on-target/off-tumor toxicity. NK cells can be independent of the host specific HLA genotype. However, in solid tumors, their efficacy remains to be fully understood. Viral transduction, transfection, mRNA electroporation and feeder-free and bovine serum-free protocol can enhance the efficacy of NK cells anticancer effects. Combination therapy using immune checkpoints and CAR NK cells can be promising for more efficient cancer therapy. The anti-KIR (lirilumab) and anti- NKG2A (monalizumab) drugs are safer than anti-PD-1 antibody and thereby can be used in combination with the CAR NK cells for melanoma treatment.

## Data Availability

Not applicable. No data has been used in this study.
